# Microbiological and Clinical Outcomes of Methicillin-Susceptible *Staphylococcus aureus* Isolated in Lung Transplant Perioperative Donor and Recipient Respiratory Cultures

**DOI:** 10.1177/09636897231182480

**Published:** 2023-07-15

**Authors:** Ghadeer Al-Ahmadi, Sagar Kothari, Hassan Almarhabi, Mohammad Bosaeed, Coleman Rotstein

**Affiliations:** 1 Immunocompromised Host Infectious Diseases Service, Ajmera Transplant Centre, University Health Network, Toronto, ON, Canada; 2 Division of Infectious Diseases, Department of Medicine, Faculty of Medicine, University of Toronto, Toronto, ON, Canada; 3 King Abdullah International Medical Research Center, King Saud Bin Abdulaziz University for Health Sciences, Riyadh, Saudi Arabia; 4 Department of Internal Medicine, King Abdulaziz Medical City, Ministry of National Guard Health Affairs, Jeddah, Saudi Arabia; 5 Department of Infectious Diseases, King Abdulaziz Medical City, Ministry of National Guard Health Affairs, Jeddah, Saudi Arabia; 6 Department of Medicine, King Abdulaziz Medical City, Ministry of National Guard Health Affairs, Riyadh, Saudi Arabia; 7 King Abdullah International Medical Research Center, College of Medicine, King Saud Bin Abdulaziz University for Health Sciences, Riyadh, Saudi Arabia

**Keywords:** *Staphylococcus aureus*, lung transplant recipients

## Abstract

*Staphylococcus aureus* is one of the most common organisms isolated from respiratory secretions in lung transplant donors and recipients perioperatively. Within the first 90 days after lung transplantation, methicillin-susceptible *Staphylococcus aureus* (MSSA) infections have been associated with increased mortality and acute and chronic rejection. However, it is unclear whether respiratory cultures positive for MSSA at the time of transplantation can lead to clinically significant infection. The aim of this study was to assess the microbiological and clinical outcomes for lung transplant recipients (LTRs) with positive perioperative donor or/and recipient respiratory cultures for MSSA. A retrospective study was conducted evaluating MSSA-positive respiratory cultures at the time of lung transplantation from donors and/or recipients from January 1, 2008, to December 30, 2019. Patients who did not have a bronchoalveolar lavage at 2 weeks after the lung transplant or died within 2 weeks of lung transplant were excluded. The main outcome was MSSA eradication at 2-week bronchoscopy. Recipients were evaluated for MSSA infections at the 12-week period after the transplant. Of the 1,678 individuals who underwent lung transplantation, 218 LTRs had *S. aureus* isolated in perioperative donor or recipient respiratory cultures, and 29 were subsequently excluded. Of the remaining 189 LTRs, MSSA eradication at the 2-week bronchoscopy was achieved in 186 (98.4%) recipients. During the 12-week follow-up, 15 (7.9%) recipients were diagnosed with MSSA pneumonia; concurrent MSSA bacteremia was noted in one recipient. No anastomotic infection, empyema, or lung abscess related to MSSA was diagnosed during the follow-up period.

In LTRs, the rate of MSSA eradication at 2-week post-transplant recipients is high, and it is associated with a low rate of infectious complication within the first 12 weeks after transplant. Most of the recipients received a combination therapy with at least one agent active against MSSA. More studies to evaluate the optimal antimicrobial stewardship policies regarding the regimen and duration of antibiotic therapy for these patients are needed.

## Introduction

Infection in lung transplant recipients (LTRs) is a leading cause of morbidity and mortality^1,2,3^. Impaired graft mucociliary function, weak cough reflex, airway ischemia, perioperative invasive procedures, and the level of immunosuppression put LTRs at increased risk of infection, particularly pneumonia^4^. During the early post-transplant period, pneumonia develops mainly due to nosocomial acquisition, recipient colonization, or donor-derived infection^5,6^. One of the most common organisms that has been implicated in positive respiratory cultures at the time of transplant is *Staphylococcus aureus*. Methicillin-susceptible *Staphylococcus aureus* (MSSA) contributes up to 25% of positive respiratory cultures in LTRs and 48% in donors^7,8^.

Methicillin-resistant *S. aureus* (MRSA) colonization at the time of lung transplant has been found to be a risk factor for progression to infection in early post-lung-transplant period, but there is no established correlation between MSSA isolated in peri-transplant respiratory culture and progression to infection^9^. At our center, piperacillin-tazobactam is used empirically for prophylaxis after lung transplantation for 72 hours until intraoperative culture results are available. The practice of most transplant physicians is to switch antibiotics to cefazolin or cloxacillin if MSSA is isolated in respiratory cultures and treat for at least 14 days with intravenous antibiotic therapy. In the case of multiple organisms being recovered, or the recipient is known to be colonized with *Pseudomonas*, cefazolin or cloxacillin is merely added to piperacillin-tazobactam. This practice is mainly derived from studies of patients with MSSA bacteremia, where using an anti-staphyloccocal penicillin or cefazolin is associated with significantly lower 30-day mortality compared to piperacillin-tazobactam^10^.

The aim of the study was to assess the rate of eradication of MSSA isolated from recipient or donor respiratory cultures at the time of lung transplantation and to assess the development of MSSA-related infection and complications during the first 12 weeks after the transplant.

## Methods

### Study Design and Patient Population

We conducted a retrospective observational cohort study at the Toronto General Hospital, a 456-bed hospital in Toronto, Ontario, Canada. LTRs with MSSA cultured from bronchoalveolar lavage (BAL) from recipients, donors, or both at the time of lung transplantation were recruited from January 1, 2008, to December 30, 2019. During the study period, the routine practice at our center consisted of performing surveillance bronchoscopy for all patients at 2 weeks, 6 weeks, and 12 weeks. All transplanted patients during the study time frame were eligible and were included if they met all the following criteria: (1) aged 18 years or older, (2) had follow-up surveillance bronchoscopy at 2 weeks, (3) survived for 2 weeks after lung transplant, and (4) if MSSA was isolated in the recipient, donor, or both. As mentioned, we categorized the source of the positive MSSA BAL culture into three categories: recipient, donor, and both donor and recipient. Patients were excluded if MRSA was cultured at the time of lung transplantation, in either the donor or recipient. The results of BAL cultures at 2 weeks, 6 weeks, and 12 weeks were assessed for MSSA eradication, persistence, recurrence, or reinfection, as defined in the following sections. Antibiotics used as a prophylaxis or treatment were assessed from the transplantation date through discharge date or 2 weeks after transplantation, whichever was longer.

### Primary and Secondary Endpoints

The primary endpoint was MSSA eradication from BAL culture at the 2-week bronchoscopy. The secondary endpoints included (1) MSSA eradication at 6 weeks and 12 weeks; (2) clinical outcome at the 2-week follow-up as assessed by clinical improvement, stability, or deterioration; and (3) clinical criteria of MSSA infection at follow-up that occurred by 12 weeks after lung transplantation (see Definitions)

### Definitions

MSSA eradication was defined as a negative BAL culture for MSSA in the follow-up bronchoscopy at 2 weeks. MSSA persistence was defined as a continuously positive BAL culture since the time of transplantation. MSSA recurrence was defined as a positive BAL culture after eradication for MSSA with the same antibiotic susceptibility testing (assumed to be the same strain) compared with the initial MSSA strain isolated at the time of transplantation. Reinfection was defined as a positive BAL culture for MSSA after eradication with a different antibiotic susceptibility (assumed to be a different strain) in comparison to the MSSA strain isolated at the time of transplantation.

The clinical assessment at 2 weeks was determined by improvement defined by a decrease in oxygen requirement, no ongoing signs of sepsis, and no new infiltration in the Chest X-ray (CXR); stability was defined by stable oxygen supplement since starting the targeted therapy, no ongoing signs of sepsis, and no new infiltration in the CXR; and deterioration by development of respiratory failure, fever or sepsis, or new infiltration in the CXR. MSSA infection within the first 12 weeks after lung transplant was defined according to the International Society for Heart and Lung Transplantation consensus criteria^11^.

### Data Collection

Data were abstracted from the database of the Toronto Lung Transplant Program of Toronto General Hospital for all patients’ cultures obtained at the time of transplantation and at 2 weeks, 6 weeks, and 12 weeks after transplantation. Paper and electronic patient charts were also examined for clinical data, laboratory data, vital signs, and diagnostic imaging. Collected data included patient demographics, underlying lung disease for lung transplant, comorbidities, antibiotic treatment, and clinical outcome.

### Statistical Analysis

Characteristics were compared using Student’s *t* test for continuous variables and chi-square or Fisher’s exact tests for categorical variables. All analyses were performed using GraphPad Prism version 9.4.1 (GraphPad Software, San Diego, CA, USA)

### Ethical Approval

The study protocol was approved by the Research Ethics Board of the University Health Network, Toronto, Ontario, Canada (REB approval number: 17-6107). Patient consent was waived for this retrospective cohort study.

## Results

### Patient Demographics and Clinical Characteristics

**Figure 1.**
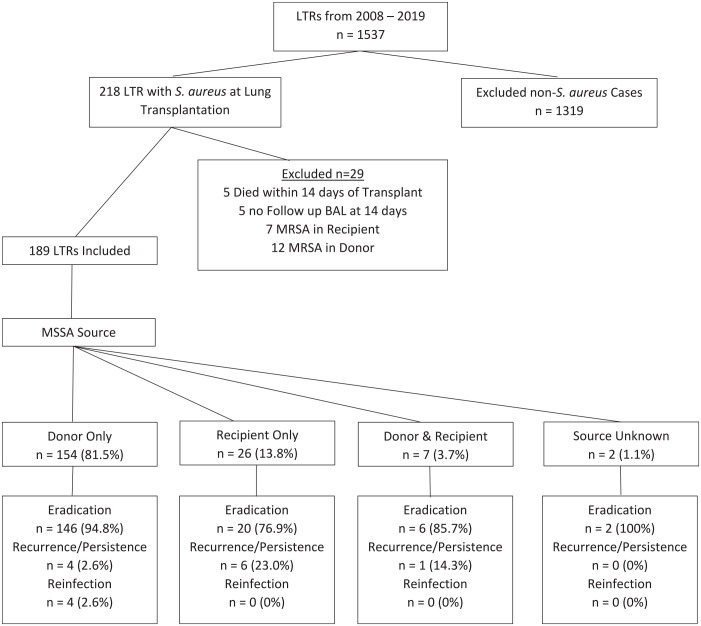
Consort diagram showing the disposition of the cohort of LTRs, 2008–2019. LTR: lung transplant recipient.

During the study period of January 1, 2008, to December 31, 2019, 1,537 patients underwent lung transplantation, of whom 218 (14.2%) had positive respiratory cultures for *S. aureus* from BAL fluid at the time of transplantation. Twenty-nine recipients were excluded due to death within 14 days of transplant (5), lack of follow-up bronchoscopy at 2 weeks (5), or isolation of MRSA (7 in the recipient and 5 in the donor) (Figure 1). The baseline demographic and clinical characteristics for the cohort of all LTRs as well as the MSSA recipients are presented in Table 1. Compared to the recipients without MSSA, the MSSA group was significantly younger and more frequently male. No differences were observed in the type of transplantation or the underlying diseases. For the MSSA group, a double-lung transplant was performed in 79.4% of the patients (150 of 189), whereas 20.6% (30 of 189) underwent a single-lung transplant. The most common indications for transplantation were interstitial lung disease (45.0%), chronic obstructive pulmonary disease and emphysema (22.8%), cystic fibrosis (19.6%), and pulmonary arterial hypertension (4.2%). The source of MSSA was donor-derived in 154 recipients (81.5%), recipient-derived in 28 recipients (14.8%), both donor- and recipient-derived in 7 recipients (3.7%), and source unknown in 2 recipients (1.1%). Co-infection with other pathogens occurred in 89 of 189 recipients, with *Pseudomonas aeruginosa* found simultaneously with MSSA in 31 patients, followed by *Haemophilus influenzae* in 24 patients, and *Enterobacteriaceae* in 20 patients.

**Table 1.** Patient Demographics and Clinical Characteristics of Lung Transplant Recipient Cohort 2008–2019.

Characteristic	N = 189	N = 1,348^a^	*P* value
Age, years, mean ± SD	51.5 ± 15.4	54.3 ± 14.2	0.01
Male sex, n (%)	111 (58.7%)	587 (43.5%)	<0.001
Diabetes mellitus, n (%)	24 (12.7%)	133 (9.9%)	0.23
Type of transplant, n (%)			
Double	150 (79.4%)	1,136 (84.3%)	0.09
Single	39 (20.6%)	212 (15.7%)	
Underlying disease, n (%)			
ILD/pulmonary fibrosis	85 (45.0%)	618 (45.8%)	0.10
Emphysema/COPD	43 (22.8%)	386 (28.6%)	
Cystic fibrosis	37 (19.6%)	171 (12.7%)	
PAH	8 (4.2%)	58 (4.3%)	
Other^b^	16 (8.5%)	115 (8.5%)	
MSSA source, n (%)			
Donor	154 (81.5%)	N/A	
Recipient	28 (14.8%)		
Donor and recipient	7 (3.7%)		
Polymicrobial infection, n (%)	89 (47%)	N/A	

BAL: bronchoalveolar lavage; COPD: chronic obstructive pulmonary disease; ILD: interstitial lung disease; MRSA: methicillin-resistant *S. aureus*; MSSA: methicillin-susceptible *Staphylococcus aureus*; N/A: not applicable; PAH: pulmonary arterial hypertension.

a Includes n = 29 with *S. aureus* found at the time of transplant: n = 5 died within 2 weeks of transplant; n = 5 had no follow-up BAL at 2 weeks after transplant; n = 7 had MRSA at the time of transplant; n = 12 had MRSA in the donor + MSSA in the recipient at the time of transplant.

b Other includes hypersensitivity pneumonitis, sarcoidosis, bronchioalveolar carcinoma, bronchopulmonary dysplasia, and retransplant for rejection.

### Treatment Characteristics

All LTRs in our study had received empirical prophylactic antibiotics after transplantation. Piperacillin-tazobactam was the most frequently administered empirical therapy (70.3%), followed by meropenem (18.5%), and then ceftazidime (8.5%). One hundred and eighteen (62.4%) recipients received at least one dose of vancomycin empirically, in addition to a beta-lactam antibiotic, prior to the culture results being finalized.

With regard to targeted antibiotic therapy for MSSA, 98 (51.9%) received a combination of antibiotics, whereas 91 (48.1%) received monotherapy. The mean duration of antibiotic therapy for MSSA was 12.5 days. The empirical and targeted antibiotic regimens for the 189 recipients are shown in Table 2.

**Table 2.** Empirical and Targeted Antibiotic Therapy for MSSA Group.

Antibiotic	N = 189
Monotherapy, n (%)	91 (48.1%)
Cefazolin or cloxacillin	69
Piperacillin-tazobactam	8
Ceftazidime	5
Meropenem	5
Vancomycin	4
Combination therapy, n (%)	98 (51.9%)
Cefazolin or cloxacillin + piperacillin-tazobactam	28
Cefazolin or cloxacillin + meropenem	19
Cefazolin or cloxacillin + quinolones	17
Dual beta-lactam (non-anti-staphylococcal agent)	12
Other^a^	22
Mean duration of targeted therapy (days ± SD)	12.5 ± 4.7

MSSA: methicillin-susceptible *Staphylococcus aureus*.

a Other include: a combination of antipseudomonal beta-lactam with either sulfamethoxazole-trimethoprim, vancomycin, linezolid, or daptomycin.

### Microbiological Outcomes

The microbiological outcomes of the 189 patients are shown in Table 3. A total of 15 patients were documented with MSSA throughout the 12-week observation period. The primary outcome of MSSA eradication at the 2-week bronchoscopy occurred in 186 (98.4%) recipients. However, at the 2-week assessment, there were 3 (1.7%) patients with documented MSSA in their respiratory secretions at the time of their bronchoscopy. In all the aforementioned MSSA isolates, the sensitivity patterns were similar to the one isolated at the time of transplant. In addition, two patients who had eradicated MSSA at the 2-week assessment were noted to have MRSA in significant numbers within 2 weeks after transplantation, verified by bronchoscopy, but this organism was not isolated at subsequent bronchoscopy assessments. At the 6-week bronchoscopy, five recipients had a positive BAL culture for MSSA (eradication in 97.3%). One recipient had a persistent positive culture at 2 weeks and 6 weeks; while the other four recipients had recurrence of MSSA after they achieved eradication at the 2-week bronchoscopy. All MSSA isolates had a susceptibility pattern similar to the one isolated at the time of transplant. Subsequently, at the 12-week bronchoscopy, seven recipients had a positive BAL culture for MSSA (96.1% eradication). Of these, four had MSSA isolates with a new susceptibility pattern suggestive of acquisition of a new MSSA isolate (reinfection), and 3 had MSSA recurrence.

**Table 3.** Microbiological Outcomes.

Outcome	
Microbiological outcome	
2 weeks, n (%)	
Eradication	186 (98.4)
Persistence	3 (1.6)
Reinfection	0 (0.0)
6 weeks, n (%)	
Eradication	168 (97.3)^a^
Recurrence	5 (2.9)
Reinfection	0 (0.0)
12 weeks, n (%)	
Eradication	167 (96.1)^b^
Recurrence	3 (1.7)
Reinfection	4 (2.3)

a Total bronchoscopies performed at 6 weeks, n = 173.

b Total bronchoscopies performed at 12 weeks, n = 174.

### Clinical Outcome

At the 2-week assessment, 150 (79.4%) recipients exhibited clinical improvement, while 29 (15.3%) recipients deteriorated, and 9 (4.8%) recipients were clinically stable. Of the 29 LTRs who deteriorated, MSSA pneumonia was confirmed in two patients. One received 14 days of vancomycin, and the second patient was on cefazolin since transplantation, which was switched to cloxacillin after a result of 2-week BAL due to progression of pneumonia. Both patients achieved full recovery. The causes of deterioration in the other 27 LTRs were attributed to non-MSSA pneumonia (14 out of 27), of which 6 were due to *P. aeruginosa* pneumonia, acute rejection (6 out of 27), or myocardial infarction (3 out of 27); while four LTRs were treated for both rejection and pneumonia. Eventually two of the patients who developed myocardial infarction died.

During the following 10 weeks of the observation period, an additional six recipients had MSSA-confirmed infection. Five of them had MSSA pneumonia, while one patient had concomitant MSSA pneumonia and bacteremia. The latter patient received 4 weeks of cefazolin for persistent MSSA bacteremia, but infective endocarditis was ruled out by transesophageal echocardiogram. Eventually, he achieved eradication of the blood stream infection and was discharged home. The other five patients received 2 weeks of different antimicrobial agents including cefazolin, piperacillin-tazobactam, and amoxicillin-clavulanic acid, and they all achieved full recovery. None of the recipients developed MSSA-related anastomotic infection, empyema, lung abscess, or infective endocarditis during the follow-up time. The details of the patient characteristics, treatment, and clinical outcomes in the recipients who developed MSSA infection are shown in Table 4.

**Table 4.** Clinical Characteristics and Outcome of Eight Patients With MSSA Infection (Pneumonia and Bacteremia) in All 12 Weeks of Assessment.

Patient	Age & gender	Underlying lung disease	Tx type	MSSA source	Peri-operative antibiotics	Duration (days)	IS regimen	Time to infection (days)	Recurrence or reinfection	Site of infection	Therapy for infection	Duration (days)	Outcome
1	42 & M	AATD	Double	Both	Vancomycin	14	CyA+MMF+Pred	14	Persistence	Pneumonia	Vancomycin	14	Recovered
2	26 & M	CF	Double	Recipient	Cefazolin	14	CyA+MMF+Pred	16	Persistence	Pneumonia	Cloxacillin	14	Recovered
3	54 & M	PF	Double	Donor	Cefazolin	14	CyA+MMF+Pred	28	Recurrence	Pneumonia	Pipracillin-tazobactam	14	Recovered
4	62 & F	ILD	Double	Donor	Cloxacillin	12	CyA+AZA+Pred	35	Reinfection	Pneumonia	Cloxacillin	14	Recovered
5	70 & M	PF	Double	Recipient	Cloxacillin	7	CyA+AZA+Pred	40	Recurrence	Pneumonia	Cefazolin	14	Recovered
6	36 & M	CF	Double	Recipient	Cloxacillin Pipracillin-tazobactam	14 21	TAC+MMF+Pred	80	Recurrence	Pneumonia and Bacteremia	Cefazolin	28	Recovered
7	51 & M	ILD	Double	Donor	Cefazolin	11	CyA+MMF+Pred	84	Recurrence	Pneumonia	Amoxacillin-clavulanic acid	14	Recovered
8	38 & M	CF	Double	Recipient	Meropenem Vancomycin	22 6	TAC+MMF+Pred	87	Recurrence	Pneumonia	Cephalexin	14	Recovered

ILD: interstitial lung disease; MSSA: methicillin-susceptible *Staphylococcus aureus*; AATD: alpha antitrypsin deficiency; CF: cystic fibrosis; PF: pulmonary fibrosis; MMF: mycophenolate mofetil; AZA: azathioprine; CyA: cyclosporine A; PRED: prednisone; IS: immunosuppression; TAC: tacrolimus.

## Discussion

We conducted a retrospective evaluation of the rate of microbiological eradication of MSSA isolated in BAL fluid at the time of transplant with follow-up to 12 weeks after lung transplant. We also looked at the development of MSSA infection and clinical outcomes in these recipients up to 12 weeks after transplant.

This study demonstrated a high rate of MSSA eradication at the 2-week post-transplant bronchoscopy in the LTRs irrespective of it being of donor or/and recipient origin. To the best of our knowledge, our study is the first of its kind evaluating the microbiological outcomes associated with MSSA isolated in the respiratory cultures of LTRs perioperatively. Interestingly, more recipients had positive respiratory cultures for MSSA at 6-week (5 of 173) and 12-week (7 of 174) bronchoscopy than at the 2-week bronchoscopy assessment (3 of 189). This could be due to the fact that most patients had been treated with antibiotics within the 2-week time frame prior to the 2-week bronchoscopy, which may have contributed to the negative respiratory cultures. At the 2-week and 6-week assessment bronchoscopies, LTRs with positive respiratory cultures were more likely to have a similar strain of MSSA than positive cultures at 12 weeks, when acquisition of new MSSA strains contributed to a 57% positivity rate of the MSSA in respiratory cultures (4 of 7). However, the overall rate of eradication remained high up to the 12-week evaluation (96.1%).

Previous studies have demonstrated that *S. aureus* is the second most common organism isolated in lung transplants perioperatively, with 74% of the isolates being MSSA.^12^ However, whether LTRs with positive MSSA cultures at the time of transplant will subsequently progress to clinically significant infection is unclear. We demonstrated a low prevalence of MSSA-related infection in those patients. Over the 12-week study period, only 8 (4.2%) recipients were diagnosed with MSSA pneumonia, and concurrent MSSA bacteremia was diagnosed in only one of these patients (0.5%). Shields et al.^9^ reported that 18% of LTRs developed *S. aureus* infection in the first 90 days after transplant, and 62% of them were due to MSSA. Although they found that the presence of MRSA in recipient sterility cultures and nasal swabs at the time of transplant was indeed a risk factor for early *S. aureus* infection, no specific risk factors were identified for MSSA infection^9^. Our low rate of infectious complications due to MSSA compared with the previous cohort may reflect the results of aggressive antibiotic management approaches taken in our LTRs from the time of transplantation. We observed that more than half of LTRs were treated with a dual antibiotic therapy. This is likely owing to the presence of polymicrobial respiratory cultures with concomitant *Pseudomonas* and other gram-negative organisms (87 of 189, 46%). It should be noted that most of the recipients who received a dual antibiotic therapy had received cloxacillin or cefazolin in combination with an anti-pseudomonal antibiotic (62 of 96, 64.6%). This practice is derived from studies on MSSA bacteremia, which indicate that treating MSSA bacteremia with piperacillin-tazobactam, vancomycin, or ceftriaxone alone was associated with a poor outcome^10,13,14^. However, there is limited evidence of the impact of using beta-lactam, other than anti-staphylococcal beta-lactam, in clinical outcomes of patients with MSSA pneumonia. A meta-analysis by Whiddon et al. found that using ceftriaxone to treat MSSA pneumonia is associated with higher clinical failure than ceftaroline or ceftobiprole, which are highly active against *S. aureus*^16^. In LTRs, such data are lacking. However, in contrary, recent data showed that antibiotic appropriateness at the time of lung transplantation has no impact on 30-day mortality, hospital length of stay, or intensive care unit length of stay^12^.

In our study, the mean duration of therapy was 12.5 days, which is considered a longer duration of antibiotic therapy. Previously, using a long-course perioperative antibiotic regimen had no mortality benefit in LTRs with positive cultures compared to a shorter course^8^. On the other hand, the duration of antimicrobials for the treatment of gram-positive infection in this population has been associated with developing drug-resistant organisms^16^. Presently, there are no studies that delineate the optimal perioperative antibiotic regimen or the duration of therapy for patients with MSSA isolated in respiratory secretions at the time of lung transplantation. More studies are needed to identify whether treatment of polymicrobial flora isolated from the BAL fluid cultures that may include MSSA at the time of transplantation requires prolonged broad-spectrum coverage, or whether a shorter duration would be adequate to diminish the threat of the emergence of resistance in keeping with the principles of antimicrobial stewardship.

Our study, however, has several limitations. The retrospective cohort design excluding patients who missed follow-up or died prior to the 2-week bronchoscopy hindered the assessment of the primary outcome and may have limited enrollment. Although none of the excluded recipients had MSSA infection as the cause of death, we still cannot comment on the short-term mortality related to MSSA due to the limited number of patients. In addition, we excluded those individuals who had positive respiratory cultures for MRSA (isolated from the donor or recipient) as well as concomitant MSSA and MRSA due to concerns about biasing the primary endpoint results for MSSA as MRSA may be harder to eradicate. Moreover, as with all retrospective studies, missing data at times posed a problem. This prevented us from comparing antibiotic regimens between those included and those excluded. Third, due to the low number of patients who received piperacillin-tazobactam or meropenem monotherapy, we could not evaluate whether the use of a broad-spectrum antibiotic alone versus an anti-staphylococcal beta-lactam may have exerted an impact on clinical or microbiological outcomes or on mortality associated with MSSA infection. Consistent with this, the number of recipients who developed clinical MSSA infection was low precluding any analysis of risk-identifying factors for MSSA infection.

In conclusion, MSSA is a commonly isolated organism in the respiratory secretion cultures of donors and recipients at the time of lung transplantation. The rate of eradication by 2 weeks was high and was associated with a low rate of infectious complication within the first 12 weeks after transplant. Most of the recipients received a combination therapy with at least one agent active against MSSA. However, it was not clear whether this is an appropriate practice or not. More studies to evaluate the optimal regimen and its duration are needed to enhance antimicrobial stewardship in LTRs.
